# Reply to Buğra Bayıcı et al.

**DOI:** 10.1093/icvts/ivag152

**Published:** 2026-05-24

**Authors:** Josías C Ríos-Ortega, Julio Morón-Castro

**Affiliations:** Cardiovascular Surgery Department, National Cardiovascular Institute, EsSalud, Lima 15072, Peru; Cardiovascular Surgery Department, National Cardiovascular Institute, EsSalud, Lima 15072, Peru

We greatly appreciate the comments on our work by Buğra Bayıcı *et al.*^1^^,^^2^ Certainly, minimally invasive cardiac surgery (MICS) has not been shown to reduce mortality or major complications (such as stroke or the need for a permanent pacemaker, etc.).^3^ However, patients undergoing MICS experience a faster recovery, less use of blood components, less chronic pain, and a quicker return to social life.^4^ These are the main advantages of MICS, in addition to its clearly better cosmetic results. One of the limitations of our study, and many others, is that postoperative pain and quality of life scales were not assessed. Future studies should address these objectives.

Based on the results of our study, we are implementing in our hospital an Enhanced Recovery protocol (which includes an ultra-fast strategy) to improve intensive care and hospital stays, not only for patients undergoing MICS but for all our patients. We expect that after implementing this protocol our results will improve.

In our study, approximately 15% of patients undergoing aortic valve replacement developed postoperative atrial fibrillation (POAF), while meta-analyses show a frequency between 11.7% and 13.3%.^4^ In fact, the aortic cross-clamp time and cardiopulmonary bypass time were significantly longer, but only by 15-20 min. We believe this had no bearing on POAF. After analysing our data, we modified our drain placement strategy. We now place a single drain outside the pericardium, over the right pulmonary hilum, leaving the pericardium partially open. This strategy is used by other groups.^5^ After using this method, we have observed a downwards trend in POAF.

## Data Availability

Authors declare the availability of data.
